# Supplementary material for: Psychological Impact of Parkinson Disease Delusions on Spouse Caregivers: A Qualitative Study
Source: Brain Sci. 2021 Jun 29;11(7):871. doi: 10.3390/brainsci11070871 (PMC8301855; doi:10.3390/brainsci11070871)
Supplement: Supplementary file 1 [file brainsci-11-00871-s001.zip › brainsci-1263414-supplementary.pdf]

PROTOCOL FOR INTERVIEW OF PWP'S SPOUSE

Name; Age; What's your education level? What's your cultural background?

How has your mental and physical health been over the last 10 years?

Can you tell me a little bit about your childhood? Your relationships with your parents, your siblings, friends, school and community?

When did you meet your spouse and how did you meet?

Could you say something about your relationship before you got married? The kinds of things you did?

How long has your spouse been diagnosed with PD?

How has your relationship been since his/her diagnosis?

How would you describe yourself in general? Your personality?

Has there been any unusual thoughts regarding you or anyone else? (give examples)

Can you describe the content of his/her delusions? Be as specific as possible with examples of what he/said.

Are you able to recall any aspect of a delusion that affected you maybe more than others?

Did you know straight away that your spouse was delusional?

Can you remember and recount when your spouse was delusional:

(a) any thoughts you had at the time?
(b) how you felt then? What emotions did you have?
(c) were you fearful of him/her talking to others about the false thoughts or embarrassed by them disclosing the false thoughts?
(d) what your outward reaction was?

Did you speak to anyone about this?

If yes – who did you speak to and how was that?

If no – was there some reason that prevented you from talking about it?

Do you think spouses should talk to someone, like a health professional, about the experience?

Did you at any point wonder about how he/she was affected by the false thoughts?

Can you talk a little about your whole experience of being with someone with PD and delusions?

How do you feel about the whole experience of caring for someone with PD and delusions? Your thoughts, your feelings and anything else that comes to mind?

What do you think is important for health teams to consider in relation to PD patients with delusions and their spouses?

Anything else you would like to mention?
